# Maternal Management of Children

**Published:** 1853-11

**Authors:** 


*Maternal Management of Children*, in Health and Disease, by Thomas Bull, M. D.. &c. Philadelphia: Lindsay & Blakiston.

This volume contains much useful information for mothers.—The chapter on medicines is especially valuable, as tending to discourage the indiscriminate use of calomel, opiates, cordials, &c., which have obtained such universal popularity in the nursery.

The book is not designed to supersede the physician, but simply to aid the mother in the management of her child in health, and in the trivial ailments. for which the nostrums of the day are used so indiscriminately and with such injurious, and in many cases fatal results to the little patients.

For sale by D. B. Cooke & Co., 185 Lake st., Chicago. J.

